# Ni-MOF/g-C_3_N_4_ S-Scheme Heterojunction for Efficient Photocatalytic CO_2_ Reduction

**DOI:** 10.3390/ma18143419

**Published:** 2025-07-21

**Authors:** Muhammad Sabir, Mahmoud Sayed, Iram Riaz, Guogen Qiu, Muhammad Tahir, Khuloud A. Alibrahim, Wang Wang

**Affiliations:** 1 State Key Laboratory of Advanced Technology for Materials Synthesis and Processing, Wuhan University of Technology, Wuhan 430070, China; m.sabir@whut.edu.cn (M.S.); qgg@whut.edu.cn (G.Q.); 2 Laboratory of Solar Fuel, Faculty of Materials Science and Chemistry, China University of Geosciences, Wuhan 430078, China; msk07@fayoum.edu.eg; 3 Chemistry Department, Faculty of Science, Fayoum University, Fayoum 63514, Egypt; 4 Department of Coatings and Polymeric Materials, North Dakota State University, Fargo, ND 58102, USA; 5 School of Mechatronical Engineering, Beijing Institute of Technology, Beijing 100081, China; 6 Department of Chemistry, College of Science, Princess Nourah bint Abdulrahman University, Riyadh 11671, Saudi Arabia; kaalibrahim@pnu.edu.sa; 7 Key Laboratory of Advanced Energy Materials Chemistry (Ministry of Education), Nankai University, Tianjin 300071, China

**Keywords:** Ni-MOF, carbon nitride, CO_2_ reduction, S-scheme heterojunction, photocatalysis

## Abstract

The rapid recombination of photoinduced charge carriers in semiconductors remains a significant challenge for their practical application in photocatalysis. This study presents the design of a step-scheme (S-scheme) heterojunction composed of carbon nitride (g-C_3_N_4_) and nickel-based metal–organic framework (Ni-MOF) to achieve enhanced charge separation. The establishment of an S-scheme charge transfer configuration at the interface of the Ni-MOF/g-C_3_N_4_ heterostructure plays a pivotal role in enabling efficient charge carrier separation, and hence, high CO_2_ photoreduction efficiency with a CO evolution rate of 1014.6 µmol g^−1^ h^−1^ and selectivity of 95% under simulated solar illumination. CO evolution represents an approximately 3.7-fold enhancement compared to pristine Ni-MOF. Density functional theory (DFT) calculations, supported by in situ irradiated X-ray photoelectron spectroscopy (XPS) and electron paramagnetic resonance (EPR) experimental results, confirmed the establishment of a well-defined and strongly bonded interface, which improves the charge transfer and separation following the S-scheme mechanism. This study sheds light on MOF-based S-scheme heterojunctions as fruitful and selective alternatives for practical CO_2_ photoreduction.

## 1. Introduction

The rapid increase in global energy consumption and the swift pace of industrialization have led to two critical challenges facing the modern world: energy shortages and environmental pollution. Addressing these issues has become an urgent priority in today’s world [1,2,3]. In recent decades, researchers have worked hard to transform CO_2_ into green chemicals, addressing ecological problems and reducing energy shortages [4,5]. Among these approaches, photoinduced CO_2_ reduction to useful chemical fuels like CO, CH_4_, and HCOOH is considered a potential strategy. The reaction reduces the concentration of atmospheric CO_2_ along with generating clean energy sources simultaneously [6,7]. However, the photocatalytic activity still remains low due to the chemical inertness and highly stable structure of CO_2_ and the poor carrier utilization efficiency of the photocatalysts [8].

On the one hand, to address the photogenerated charge carriers’ recombination issue, S-scheme heterojunction photocatalysts with adequate ability to spatially separate photogenerated electron–hole pairs were proposed and demonstrated to achieve higher photocatalytic performance [9,10,11]. An S-scheme heterojunction is established by coupling a reduction photocatalyst (RP) with an oxidation photocatalyst (OP). The disparity in their work functions induces the formation of an internal electric field (IEF) and band bending at the interface. This facilitates the migration of photoexcited electrons in the OP and holes in the RP to the heterointerface, where recombination occurs. Consequently, electrons are retained in the RP, and holes remain in the OP, both exhibiting strong redox potentials [12,13,14,15,16,17]. Thus, S-scheme heterojunctions improve the redox capabilities of photocatalysts by utilizing band bending and the IEF, leading to enhanced photocatalytic activity [18].

On the other hand, variety of products can be produced during photocatalytic CO_2_ reduction due to the complex elementary steps and electron transfer pathways involved [19]. Therefore, improving the system activity and selectivity toward a target product is a general pursuit of CO_2_ photoreduction research terrain [20,21]. In this vein, a diverse array of photocatalytic materials have been employed in order to enhance CO_2_ photoreduction performance including metal nanoparticles [22,23], metal oxides [24,25], and metal–organic frameworks (MOFs) [26,27]. Among them, MOFs have emerged as a highly promising class of materials due to their unique structure. Composed of metal ions coordinated with organic ligands [28,29,30], MOF-based photocatalysts possess a large specific surface area and high porosity, which enables efficient adsorption of reactants [31,32]. Furthermore, the tailored structure of MOFs can induce preferential adsorption and activation of CO_2_ molecules, favoring the activity and selectivity of the photoreduction [33,34].

Alternately, graphitic carbon nitride (g-C_3_N_4_) is a well-known metal-free n-type semiconductor with an ultrathin two-dimensional (2D) structure, exhibiting outstanding physicochemical properties. The properties of g-C_3_N_4_ (CN), such as its non-toxicity, ease of synthesis, and abundant availability, establish it as a promising material for various applications in both industrial and practical contexts. CN has garnered significant attention in the field of photocatalysis due to its appropriate band gap, easily tunable structure, and exceptional chemical and thermal stability [35,36]. However, due to its rapid electron–hole recombination and limited light absorption capacity, CN is often combined with other materials to form heterojunctions, enhancing its photocatalytic efficiency instead of functioning independently [37].

Herein, an S-scheme heterojunction composed of a nickel-based MOF (Ni-MOF) and CN was synthesized using a wet chemical approach for enhanced and selective photocatalytic CO_2_ reduction. Under 300 W Xe lamp irradiation, the synthesized Ni-MOF/CN composite demonstrates a superior and selective CO production compared to pure Ni-MOF. The charge carrier transfer pathway in the Ni-MOF/CN S-scheme heterojunction was elucidated through in situ XPS, EPR, and DFT calculations. The high activity of the Ni-MOF/CN S-scheme heterojunction provides proof of its potential as an effective and selective photocatalyst for CO_2_ photoreduction.

## 2. Materials and Methods

### 2.1. Material

All chemical reagents were of analytical grade and utilized without additional purification. Dicyandiamide (CN) (99%) and terephthalic acid (99%) were obtained from Aladdin (Shanghai, China). Nickel (II) nitrate hexahydrate and polyvinylpyrrolidone (K30) were sourced from Sinopharm Chemical Reagent Co., Ltd. (Shanghai, China), along with ethanol (99.8%), acetonitrile (99.5%), and triethanolamine (99%). Deionized water (DI) was employed throughout the experimental procedures.

### 2.2. Synthesis of Photocatalyst

#### 2.2.1. Synthesis of Ni-MOF

A homogeneous solution was prepared by mixing 20 mL of deionized water, 20 mL of ethanol, and 20 mL of DMF in a 100 mL beaker under continuous stirring for 10 min. Subsequently, 1.6 g of nickel (II) nitrate hexahydrate (Ni(NO_3_)_2_·6H_2_O), 0.48 g of terephthalic acid, and 2.4 g of polyvinylpyrrolidone (PVP, K30) were added to the solution, followed by vigorous stirring for 30 min to ensure thorough dissolution and uniform dispersion. The reaction mixture was sealed in a 100 mL Teflon-lined autoclave and heated at 150 °C for 10 h under hydrothermal conditions. After natural cooling, the precipitate was collected by centrifugation, purified through repeated ethanol and DI water washes, and dried to yield the final product as a light green powder.

#### 2.2.2. Synthesis of CN

CN nanosheets were fabricated through a two-step thermal oxidation etching process. Initially, bulk CN was prepared by calcining 10 g of dicyandiamide in a covered alumina crucible using a muffle furnace. The precursor was heated to 550 °C at a ramp rate of 2.5 °C/min and maintained at this temperature for 4 h under ambient conditions. Following thermal treatment, a yellow agglomerate was obtained, which was manually ground into a fine powder using a mortar and pestle. To exfoliate the bulk CN into ultrathin nanosheets, 0.1 g of the powdered sample was subjected to secondary calcination in an open ceramic boat. The temperature was raised to 510 °C at a heating rate of 5 °C/min and held for 2 h. This step induced thermal etching, leading to the formation of few-layered CN nanosheets with enhanced surface area and reduced thickness.

#### 2.2.3. Synthesis of Ni-MOF/CN

**Figure 1:**
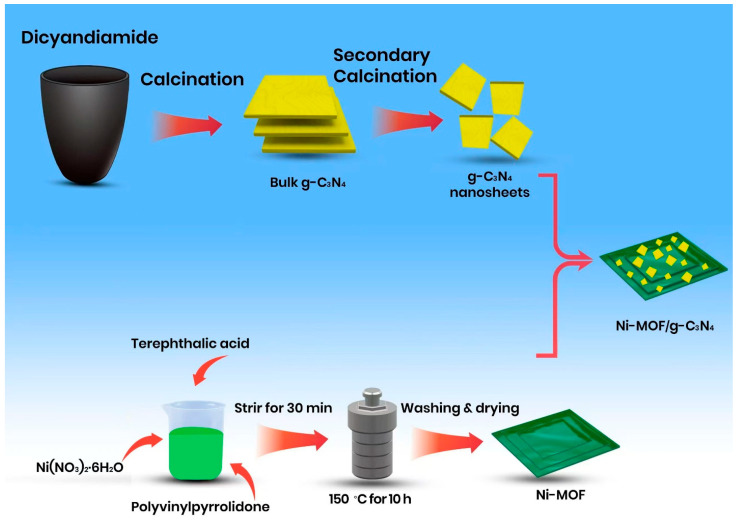
Schematic diagram for Ni-MOF/CN heterostructure synthesis.

All the samples were prepared through the wet chemical method by directly dispersing Ni-MOF and CN into a 20 mL mixture (1:1) of ethanol and water. The mixtures were subjected to continuous stirring followed by ultrasonication for 30 min to ensure homogeneous dispersion. After sonication, the samples were filtered and dried at 80 °C (Figure 1). The resulting composites were designated as CN/NMF-1, CN/NMF-2, CN/NMF-3, CN/NMF-4, CN/NMF-5, and CN/NMF-6, corresponding to Ni-MOF loadings of 20, 40, 60, 80, 85, and 90 wt%, respectively.

During the synthesis of Ni-MOF, polyvinylpyrrolidone (PVP) was introduced as a capping and structure-directing agent. PVP selectively adsorbs onto crystal facets, controlling the nucleation and growth of Ni-MOF and promoting the formation of uniform 2D nanosheets with minimal aggregation.

## 3. Results and Discussion

### 3.1. Phase Composition and FT-IR Analysis

**Figure 2:**
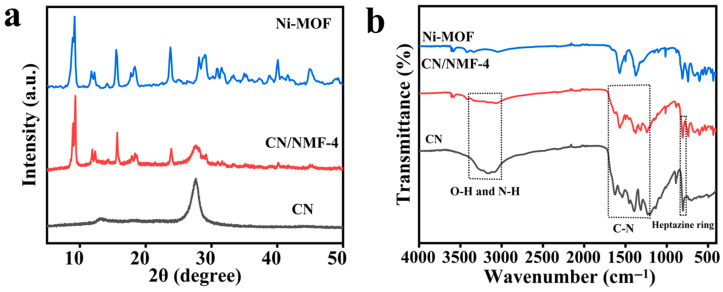
(**a**) XRD patterns; (**b**) FT-IR patterns.

The X-ray diffraction (XRD) patterns of CN, Ni-MOF, and CN/NMF-4 samples are presented in Figure 2a. Ni-MOF exhibited well-defined, sharp, and distinct peaks, indicating the high crystallinity of the synthesized Ni-MOF. The peaks at 9.2°, 11.8°, 15.6°, 18.2°, 23.7°, 28.0°, 34.9°, 40.1°, and 45.1° correspond well to the (100), (010), (101), (200), (020), (121), (221), (331) and (510) crystal planes, respectively [38]. For CN, the diffraction peaks at 13.0° and 27.7° are attributed to the (100) and (002) planes of CN, respectively [39]. For CN/NMF-4, clear diffraction peaks from both components are observed, indicating strong interaction between CN and Ni-MOF. The XRD results validate the successful preparation of the CN/NMF composites. Although nickel hydride (NiH) formation may occur under elevated temperatures or strongly reducing conditions, our synthesis was performed under mild solvothermal conditions without reducing agents. Moreover, XRD and XPS analyses show no evidence for NiH species, confirming that nickel remains as Ni^2+^ within the Ni-MOF framework. Therefore, the presence of NiH in the final material is unlikely and does not impact the photocatalytic CO_2_ reduction performance [40].

Fourier-transform infrared (FT-IR) spectroscopy was utilized to analyze the functional groups in Ni-MOF, CN, and the CN/Ni-MOF-4 samples, as shown in Figure 2b. In the Ni-MOF spectrum, the vibrational peak observed at 720 cm^−1^ corresponds to the out-of-plane bending mode of the benzene ring within the substituted aromatic structure [41]. Additionally, the absorption bands at 1100 cm^−1^ arise from C-H bending vibrations of the benzene ring [42]. The intense peaks at 1575 and 1373 cm^−1^ are linked to the asymmetric and symmetric stretching vibrations of the -COOH groups [43,44]. In the CN spectrum, the characteristic absorption peak at 808 cm^−1^ is attributed to the N-C=N vibrational mode within the heptazine ring framework. Furthermore, the multiple peaks in the range of 1200–1700 cm^−1^ originate from C-N stretching vibrations. A broad absorption band in the range of 3000–3400 cm^−1^ suggests the presence of terminal amine (-NH_2_) or hydroxyl (-OH) groups [45]. The composite samples show the distinctive signals of both CN and Ni-MOF, further demonstrating the effective integration of CN and Ni-MOF in a heterostructure configuration.

### 3.2. Morphological Characterizations

**Figure 3:**
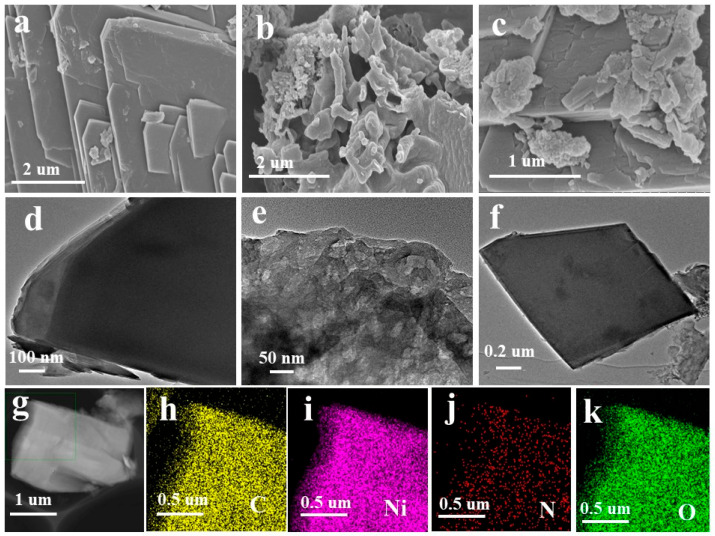
FESEM images of (**a**) Ni-MOF, (**b**) CN, and (**c**) CN/NMF-4; TEM images of (**d**) Ni-MOF, (**e**) CN, and (**f**) CN/NMF-4; (**g**–**k**) elemental mapping of CN/NMF-4.

The morphologies of Ni-MOF, CN, and CN/NMF-4 were investigated using field-emission scanning electron microscopy (FESEM) and transmission electron microscopy (TEM), as depicted in Figure 3. Ni-MOF exhibited a well-defined and uniformly stacked 2D nanosheet morphology, as shown in Figure 3a,d, and Figure S1 (Supporting Information). Also, pure CN displayed an ultrathin 2D nanosheet structure (Figure 3b,e). The 2D/2D configuration of Ni-MOF/CN structure endows them with promising merits such as (i) strong interfacial interaction for better charge separation/migration and (ii) strong electronic coupling between 2D layers, which optimizes the electronic structure of the composite material for upgraded photocatalytic activity. After CN integration with Ni-MOF, CN sheets were successfully deposited onto the 2D layers of Ni-MOF (Figure 3c,f; Figure S2, Supporting Information). The successful deposition of CN onto Ni-MOF hints at the creation of a strong heterojunction between CN and Ni-MOF. Additionally, elemental mapping (Figure 3g–k) revealed the existence of carbon (C), nickel (Ni), nitrogen (N), and oxygen (O), hence validating the CN/NMF-4 composite composition. Thermal stability of pure Ni-MOF, CN, and CN/NMF-4 was evaluated through thermal gravimetric analysis, (Supporting Information, Figure S3 and Table S3).

### 3.3. Photocatalytic CO_2_ Reduction Performance

The photocatalytic CO_2_ reduction reactions were conducted in a mixed acetonitrile-water solution under simulated solar light illumination. Ru(bpy)_3_Cl_2_ was used as the photosensitizer and triethanolamine was used as the electron donor. Before evaluating the photocatalytic performance of Ni-MOF and its composites for CO_2_ reduction, a series of control experiments under different conditions, including in the absence of CO_2_ gas, without the photocatalyst, and without light irradiation, were conducted. In all these cases, no detectable products were observed, which confirms that the observed CO formation is exclusively attributed to the photocatalytic CO_2_ reduction process facilitated by the prepared photocatalytic materials. Under the reaction conditions, Ru(bpy)_3_Cl_2_ photosensitizer shows no activity for CO_2_ reduction when tested as a photocatalyst in the absence of Ni-MOF or its composite samples. Therefore, it is rational to assign a cocatalytic role solely for Ru(bpy)_3_Cl_2_. This cocatalytic role can involve the injection of excited electrons into the conduction band of the confined semiconductor to further improve the CO_2_ activation process.

Alternately, CN/NMF-4 exhibited a very low CO production rate (5.6 µmol g^−1^ h^−1^) without adding Ru(bpy)_3_Cl_2_. Therefore, we believe that the prepared samples of ca. Ni-MOF and its composite with CN provide a functional substrate for CO_2_ adsorption and subsequent activation. Notably, the CO yield significantly increased upon the integration of Ru(bpy)_3_Cl_2_, clearly demonstrating the synergistic interaction between the sensitizer and the heterojunction photocatalyst.

**Figure 4:**
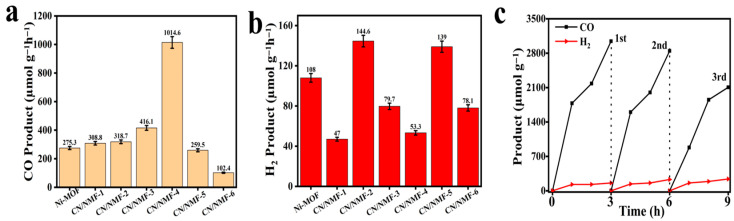
(**a**) CO evolution rate of all the samples; (**b**) H_2_ evolution rate of all the samples; (**c**) recycling experiments of CN/NMF-4.

The photocatalytic CO production performance of Ni-MOF and CN/Ni-MOF composites is presented in Figure 4a. All experiments were performed at room temperature. An image of the on-line photocatalytic CO_2_ reduction setup is provided in Figure S4 (Supporting Information). The CO evolution rate of pure Ni-MOF photocatalyst is 275.1 μmol g^−1^ h^−1^. After introducing CN into Ni-MOF, the CO production significantly increases for the CN/Ni-MOF composites. The CO_2_ reduction activity seems to align partly with the Ni-MOF content. The composite sample CN/NMF-4 exhibits the highest photocatalytic CO_2_ reduction activity, achieving an average CO production rate of 1014.6 μmol g^−1^ h^−1^, which is 3.7 times higher than that of pure Ni-MOF. However, further increasing the Ni-MOF content resulted in a decreased CO_2_ reduction activity. This can be attributed partly to the blockage of active sites with increasing Ni-MOF content. Another reason for the decline in activity is the limited heterojunction interface that is constructed between non-equivalent compositions of CN and Ni-MOF, which culminates in poor photogenerated charge carrier separation and hence limited activity.

Furthermore, the activity of the prepared samples was tested for the hydrogen evolution reaction (HER), as a potent competitive reaction. The H_2_ production rate of the CN/NMF-4 sample reaches 53.3 μmol g^−1^ h^−1^ (Figure 4b), indicating a CO production selectivity of ~95%.

Different parameters can affect the CO_2_ reduction reaction selectivity, including catalyst composition, catalyst electronic and optical features, and catalyst surface properties, among others [37]. The first step in the CO_2_ and HER reduction half-reactions represent adsorption of reactant molecules (i.e., CO_2_ and H^+^, respectively) at active sites. Therefore, it is safe to assume that CN/NMF composite heterostructures provide more favorable adsorption sites for CO_2_ molecules over H^+^ ions. Apart from the adsorption of reactant molecules onto the surface, different thermodynamic and kinetic factors will then influence the subsequent activation steps. Therefore, the HER activity of the prepared samples and hence the CO_2_ reduction selectivity do not show a regular trend. However, it is clear that the optimum CN/NiMOF ratio can promote the CO_2_/CO reaction and suppress the HER, thus improving the selectivity for CO generation.

The enhanced CO production rate of the Ni-MOF/CN composite is comparable to that of representative Ni-MOF-based and composite photocatalysts, as shown in Table S1 (Supporting Information). An excessive amount of Ni-MOF appears to suppress CO production. The cycling stability of the prepared catalysts was evaluated (Figure 4c). Even after three consecutive cycles, CN/NMF-4 demonstrated good performance, with a slightly decreased activity. XRD and FTIR measurements for the spent CN/NMF-4 photocatalyst were performed to check the catalyst stability and/or possible structural rearrangement. The results are compared with the fresh catalyst sample. As shown in Figures S6 and S7, it is confirmed that no structural or compositional change occurs after the catalytic reaction, indicating structural robustness. The activity decline can be attributed to catalyst poisoning by CO after consecutive activity cycles.

### 3.4. UV–Vis Diffuse Reflectance Absorption Analysis (DRS) and the Mott–Schottky Plots

**Figure 5:**
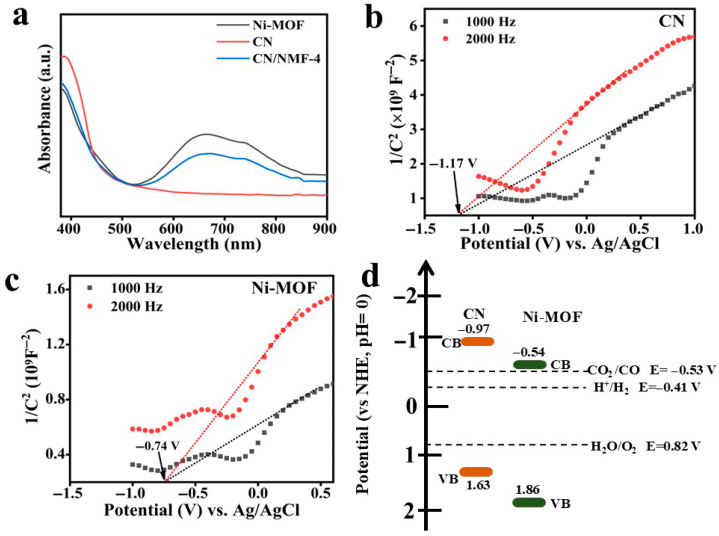
(**a**) UV–vis DRS spectra of as-prepared samples. The Mott−Schottky plot of CN (**b**) and Ni-MOF (**c**). (**d**) Band structure diagram of CN and Ni-MOF.

The optical properties of pure Ni-MOF, CN, and CN/NMF-4 were investigated using UV–vis diffuse reflectance spectroscopy (Figure 5a). Ni-MOF demonstrates a broad absorption spectrum within the visible light region with two distinct peaks, one corresponding to its intrinsic absorption band edge within the 400–500 nm range and the other broad absorption peak observed between 600 and 800 nm. This latter peak can be attributed to charge transfer transitions between oxygen 2p orbitals and Ni^2+^ 3d orbitals [46]. The absorption edge of CN is observed at approximately 470 nm. Interestingly, the CN/NMF-4 nanocomposites demonstrate enhanced absorption in the visible region. A higher light absorption ability leads to the generation of a greater number of electron–hole pairs, and hence, better photocatalytic performance would be achieved. The band gaps of CN and Ni-MOF were calculated using Tauc’s equation, with the obtained values being 2.6 eV and 2.4 eV, respectively (Supporting Information, Figure S5).

The evaluation of energy band positions represents a fundamental aspect in elucidating photocatalytic reaction mechanisms. In the present investigation, flat-band potentials were established through the Mott–Schottky measurements performed at multiple frequencies. The characteristic plots displayed in Figure 5b,c demonstrate positive linear regions for both CN and Ni-MOF, confirming their n-type semiconducting nature. Quantitative analysis revealed flat-band potentials of CN and Ni-MOF to be −1.17 V and −0.74 V (vs Ag/AgCl, pH = 7), respectively. Using the relation E_NHE_ = E_Ag_/_AgCl_ + 0.197 [47], the flat-band potentials correspond to −0.97 V for CN and −0.54 V for Ni-MOF (vs. NHE, pH = 0). Considering the well-documented approximation that the conduction band edge potential in n-type semiconductors closely aligns with the flat-band potential [48], the CB potentials of CN and Ni-MOF were calculated to be −0.97 V and −0.54 V vs. NHE, respectively. Subsequent determination of VB potentials employed the fundamental relationship *E*_VB_ = *E*_g_ + *E*_CB_ [49], where *E*_g_ represents the optical bandgap, resulting in calculated VB positions of +1.63 V for CN and +1.86 V for Ni-MOF. The comprehensive band alignment diagram derived from these analyses is presented in Figure 5d.

### 3.5. DFT Calculation

**Figure 6:**
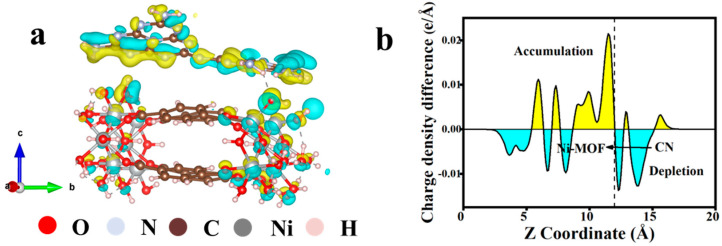
(**a**) Three-dimensional and (**b**) planar-averaged charge density differences of CN/NMF-4 heterojunction (electron accumulation and depletion are marked by the yellow and cyan colors, respectively).

DFT calculations were utilized to investigate the interfacial charge transfer behavior between CN and Ni-MOF. The work functions were utilized to elucidate the direction of charge transfer and the band alignments within the heterojunction. When CN is combined with Ni-MOF, the difference in their work functions facilitates the flow of charges or electrons from CN to Ni-MOF. An interfacial electric field directed from CN to Ni-MOF is induced until the Fermi levels of the two materials reach equilibrium. This is depicted in Figure 6a,b where the three-dimensional and planar-averaged charge density differences clearly reveal significant electron transfer at the CN/Ni-MOF interface. Electrons are depleted near the CN layer and they accumulate on the surface of Ni-MOF, ultimately generating an interfacial electric field directed from CN to Ni-MOF.

### 3.6. XPS and EPR Analysis

**Figure 7:**
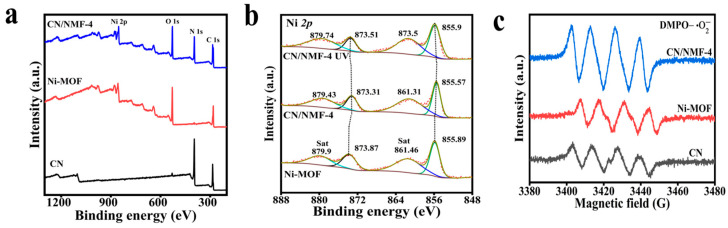
(**a**) XPS survey patterns and (**b**) XPS spectra of Ni *2p* of Ni-MOF and CN/NMF-4; (**c**) EPR spectra for DMPO-**^.^**O_2_**^–^**.

Furthermore, XPS was utilized to analyze the chemical states of the constituent elements in the synthesized materials and to explore the charge transfer processes within the CN/Ni-MOF composites. The survey spectra presented in Figure 7a clearly identify the surface elemental composition of pristine CN, Ni-MOF, and the CN/NMF-4 composite. The spectrum of the CN/NMF-4 sample shows distinct peaks for C, N, Ni, and O, indicating the successful synthesis of both the CN and Ni-MOF components. High-resolution Ni *2p* spectra (Figure 7b) are presented for both pure Ni-MOF and CN/NMF-4. In the Ni-MOF spectrum, Ni *2p* peaks are observed at binding energies of 855.89 eV and 873.87 eV, corresponding to Ni *2p*_3/2_ and *2p*_1/2_ peaks, respectively [50]. Distinct shifts to lower binding energies (BEs) are observed for the Ni *2p* peaks in the CN/NMF-4 sample compared to pristine Ni-MOF. This finding clearly indicates a charge transfer from CN to Ni-MOF upon contact. Under light irradiation, however, the trend is reversed, with slight positive shifts observed for the characteristic peaks of Ni *2p*. This suggests a directional electron transfer from Ni-MOF to CN. These findings give strong evidence that the charge transfer dynamics in the CN/NMF-4 composite adhere to an S-scheme configuration [51,52].

EPR spectroscopy was conducted to examine the charge transfer behavior in the sample. As shown in Figure 7c, the EPR spectra of DMPO-**^.^**O_2_^–^ adducts were recorded for CN, Ni-MOF, and CN/NMF-4. The CN/NMF-4 composite exhibited significantly enhanced EPR signals compared to the individual components, suggesting a more effective separation of photogenerated charge carriers. This enhancement is attributed to the S-scheme heterojunction formation, which facilitates the retention of photogenerated electrons in the conduction band of CN. These electrons are subsequently involved in the reduction of molecular oxygen (O_2_) to superoxide radicals (**^.^**O_2_^–^.). Thus, the EPR findings further corroborate the presence and functionality of the S-scheme charge transfer mechanism in the CN/NMF-4 system.

### 3.7. Photoluminescence and Electrochemical Analysis

**Figure 8:**
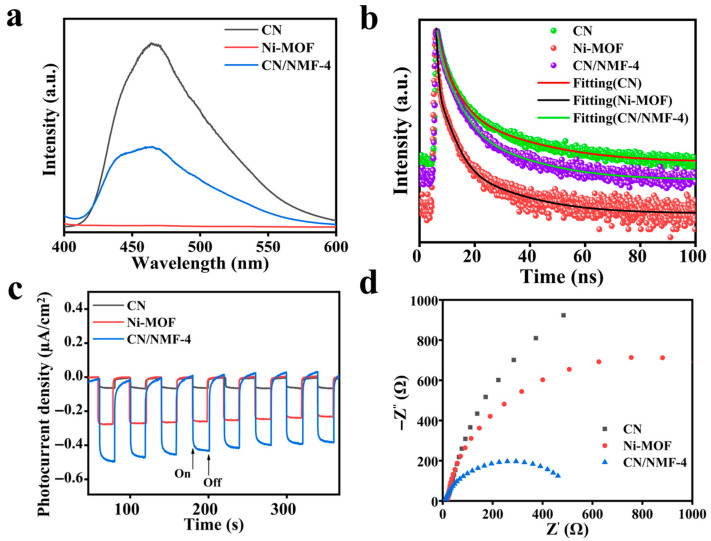
Photoluminescence spectroscopy (**a**), fitting of time-resolved photoluminescence spectra (**b**), photocurrent response (**c**), and EIS measurement (**d**) for CN/NMF, CN, and Ni-MOF samples.

The efficiency of electron separation and transfer is a key factor influencing the overall photocatalytic performance. The photoluminescence (PL) spectra of the composite sample and individual components are given in Figure 8a. The mean emission peak of pure CN is centered around 460 nm, corresponding to band-to-band charge recombination. It is extensively reported that pure CN exhibits a very strong recombination tendency for photogenerated carriers. After compositing CN with Ni-MOF, the intensity of PL peak is drastically suppressed. This reduction is directly associated with the enhanced separation of photogenerated charge carriers resulting from the formation of the CN/NMF-4 heterostructure. This observation suggests that the composite samples exhibit improved charge carrier separation efficiency, which contributes to enhanced photocatalytic activity. The time-resolved photoluminescence (TRPL) decay profiles shown in Figure 8b offer critical insights into the charge carrier separation and recombination kinetics governing the S-scheme heterojunction mechanism [53]. TRPL analysis revealed distinct average photoluminescence lifetimes of 8.36, 3.65, and 6.01 ns for CN, Ni-MOF, and CN/NMF-4, respectively, with comprehensive lifetime fitting parameters provided in Table S1 (Supporting Information). CN/NMF-4 possesses a markedly shorter average lifetime than CN, indicating more efficient migration of photogenerated charge carriers. This enhancement in carrier transport results in a reduced fluorescence lifetime.

Photoelectrochemical characterization was conducted to evaluate the enhanced charge carrier in the synthesized materials. As shown in Figure 8c, comparative analysis of transient photocurrent responses reveals that the CN/NMF-4 heterostructure achieves higher photocurrent density than pristine CN and Ni-MOF, demonstrating significantly improved charge separation efficiency through heterojunction formation [54]. Complementary electrochemical impedance spectroscopy (EIS) serves as a powerful analytical technique for evaluating charge carrier transport dynamics across the electrode–electrolyte interface. The CN/NMF-4 electrodes exhibited the smallest arc radius in the EIS Nyquist plot, indicating enhanced charge transfer at the electrode interface compared to the other samples, as depicted in Figure 8d. The reduction in the diameter of the semicircle suggests a decrease in resistance and an improvement in the mobility of charge carriers toward the interface. These findings highlight that the formation of the S-scheme heterojunction between CN and Ni-MOFs significantly boosts interfacial charge transfer and separation [55,56].

**Figure 9:**
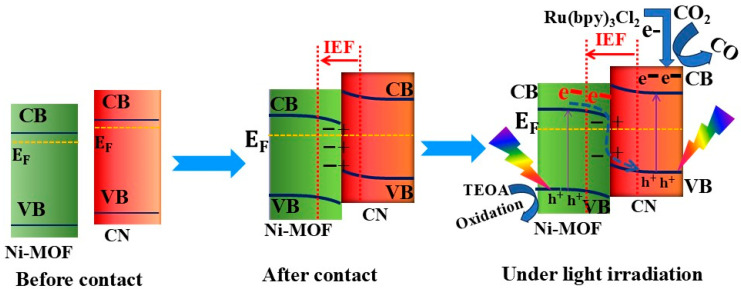
Schematic of work function alignment before and after contact and the S-scheme charge transfer mechanism in the Ni-MOF/CN heterojunction under light irradiation. Electron migration establishes an internal electric field, promoting charge separation and enabling efficient CO_2_ reduction and oxidation reactions.

To elucidate the enhanced photocatalytic activity of the CN/NMF-4 composite, a mechanism based on an S-scheme charge transfer was proposed, as depicted in Figure 9. Prior to junction formation, Ni-MOF and CN exhibit distinctly different Fermi levels. Upon contact, electrons migrate from CN to Ni-MOF until their Fermi levels equilibrate, generating an internal electric field (IEF) at the interface. This field plays a pivotal role in facilitating charge separation and directing charge carrier movement. Upon illumination, both materials absorb photons and generate electron–hole pairs. Due to the established IEF and band alignment, electrons in the CB of Ni-MOF preferentially recombine with holes in the VB of CN. This recombination mechanism characterizes the S-scheme configuration, where the most reactive electrons in CN and holes in Ni-MOF are spatially retained. As a result, photogenerated electrons in the CB of CN drive CO_2_ reduction to CO, while the holes in the VB of Ni-MOF participate in oxidative reactions. This spatial separation of redox-active species effectively suppresses electron–hole recombination, extends carrier lifetimes, and enhances the redox capacity of the system, culminating in superior photocatalytic CO_2_ conversion performance.

## 4. Conclusions

A straightforward wet chemical method was employed to fabricate an S-scheme heterojunction between Ni-MOF and CN. The formation of the heterojunction and the strong interfacial contact between the components were confirmed through comprehensive analytical techniques. FESEM and TEM analyses demonstrated the uniform distribution of CN on the Ni-MOF surface. The optimized CN/NMF-4 composite photocatalyst exhibited high CO_2_ photoreduction performance, achieving a CO production rate of 1014.6 µmol g^−1^ h^−1^ with a high CO selectivity of 95% under simulated solar illumination for CN/NMF-4, which is approximately 3.7 times higher than that of pure Ni-MOF. The enhanced photocatalytic performance was ascribed to the improved separation and mobility of photogenerated charge carriers, driven by the well-constructed interfacial contact in the S-scheme heterojunction. Comprehensive analyses involving DFT calculations, in situ XPS, and EPR results provided conclusive evidence for the efficient interfacial charge transfer between Ni-MOF and CN upon heterojunction formation and light irradiation, corroborating the proposed S-scheme charge migration pathway. This study offers a promising strategy for designing heterojunctions supported by metal–organic frameworks to achieve targeted control of charge carrier dynamic for efficient and selective CO_2_ photoreduction.

## Data Availability

The original contributions presented in this study are included in the article/Supplementary Materials. Further inquiries can be directed to the corresponding authors.

## Supplementary Materials

The following supporting information can be downloaded at https://www.mdpi.com/article/10.3390/ma18143419/s1. Figure S1. FESEM of Ni-MOF. Figure S2. FESEM of CN/NMF. Figure S3. TGA analysis of Ni-MOF, CN, and CN/NMF-4. Figure S4. Image of the on-line photocatalytic CO_2_ reduction system. Figure S5. Tauc’s plot of Ni-MOF and CN. Figure S6. XRD. Figure S7. FTIR, after and before the reaction. Table S1: Ni-MOFs and their composite-based photocatalysts for CO_2_ reduction. Table S2: Fitting parameters of time-resolved photoluminescence spectra of CN, Ni-MOF, and CN/NMF-4 samples. Table S3: Thermal gravimetric analysis (TGA) data. References [42,47,50,57,58,59,60,61,62,63,64,65,66,67,68,69,70,71] are cited in Supplementary Materials.

## References

[1] Sayed M., Yu J., Liu G., Jaroniec M. (2022). Non-Noble Plasmonic Metal-Based Photocatalysts. Chem. Rev..

[2] Li C., Lu X., Chen L., Xie X., Qin Z., Ji H., Su T. (2024). WO_3_/BiOBr S-Scheme Heterojunction Photocatalyst for Enhanced Photocatalytic CO_2_ Reduction. Materials.

[3] Xu H., Song H., Wang X., Zhu X. (2025). Oxygen Vacancy Modification Mil-125 (Ti) Promotes CO_2_ Photoreduction to CO with near 100% Selectivity. Materials.

[4] Sayed M., Xu F., Kuang P., Low J., Wang S., Zhang L., Yu J. (2021). Sustained CO_2_-Photoreduction Activity and High Selectivity over Mn, C-Codoped ZnO Core-Triple Shell Hollow Spheres. Nat. Commun..

[5] Fang Z., Ge H., Lu Y., Liu X., Zhang Z. (2025). Preparation, Stability, and Enhanced CO_2_ Absorption and Desorption of Nanofluids: Review and Perspectives. J. Environ. Chem. Eng..

[6] Dong W.-W., Jia J., Wang Y., An J.-R., Yang O.-Y., Gao X.-J., Liu Y.-L., Zhao J., Li D.-S. (2022). Visible-Light-Driven Solvent-Free Photocatalytic CO_2_ Reduction to CO by Co-MOF/Cu_2_O Heterojunction with Superior Selectivity. Chem. Eng. J..

[7] Cho J., Medina A., Saih I., Il Choi J., Drexler M., Goddard W.A., Alamgir F.M., Jang S.S. (2024). 2d Metal/Graphene and 2d Metal/Graphene/Metal Systems for Electrocatalytic Conversion of CO_2_ to Formic Acid. Angew. Chem..

[8] Wu H.L., Li X.B., Tung C.H., Wu L.Z. (2019). Semiconductor Quantum Dots: An Emerging Candidate for CO_2_ Photoreduction. Adv. Mater..

[9] He F., Zhu B., Cheng B., Yu J., Ho W., Macyk W. (2020). 2d/2d/0d TiO_2_/C_3_N_4_/Ti_3_C_2_ Mxene Composite S-Scheme Photocatalyst with Enhanced CO_2_ Reduction Activity. Appl. Catal. B Environ..

[10] Sayed M., Qi K., Wu X., Zhang L., García H., Yu J. (2025). Cu-Based S-Scheme Photocatalysts. Chem. Soc. Rev..

[11] Liu X., Peng X., Fu T., Shen C., Ding K., Li J., Yang Y., Lin H., Liu Z., Hu A. (2025). A Comprehensive Review of S-Scheme Heterojunction Photocatalysts for CO_2_ Reduction: Design Principles, Mechanisms, and Material Classification. J. CO_2_ Util..

[12] Wang L., Fei X., Zhang L., Yu J., Cheng B., Ma Y. (2022). Solar Fuel Generation over Nature-Inspired Recyclable TiO_2_/g-C_3_N_4_ S-Scheme Hierarchical Thin-Film Photocatalyst. J. Mater. Sci. Technol..

[13] Xu F., Meng K., Cao S., Jiang C., Chen T., Xu J., Yu J. (2021). Step-by-Step Mechanism Insights into the TiO_2_/Ce_2_S_3_ S-Scheme Photocatalyst for Enhanced Aniline Production with Water as a Proton Source. ACS Catal..

[14] Zhang L., Wang Z.-Q., Liao J., Zhang X., Feng D., Deng H., Ge C. (2022). Infrared-to-Visible Energy Transfer Photocatalysis over Black Phosphorus Quantum Dots/Carbon Nitride. Chem. Eng. J..

[15] Liang T., Yu Z., Bin Y., Zhang S., Wei J., Liu Y., Zhu T., Fan S., Shen Y., Wang S. (2024). Tungsten and Oxygen Dual Vacancies Regulation of the S-Scheme ZnSe/ZnWO_4_ Heterojunction with Local Polarization Electric Field for Efficient CO_2_ Photocatalytic Reduction. Chem. Eng. J..

[16] Cheng S., Sun Z., Lim K.H., Wibowo A.A., Zhang T., Du T., Liu L., Nguyen H.T., Li G.K., Yin Z. (2023). Dual-Defective Two-Dimensional/Two-Dimensional Z-Scheme Heterojunctions for CO_2_ Reduction. ACS Catal..

[17] Hu C., Cao J., Jia X., Sun H., Lin H., Chen S. (2023). Difunctional Ni_2_P Decorated Novel Z-Scheme BiVO_4_/g-C_3_N_4_ Heterojunction for Achieving Highly Efficient CO_2_ Reduction and Tetracycline Oxidation. Appl. Catal. B Environ..

[18] Cheng J., Cheng B., Xu J., Yu J., Cao S. (2024). Organic–Inorganic S-Scheme Heterojunction Photocatalysts: Design, Synthesis, Applications, and Challenges. eScience.

[19] Li C., Wang J., Tong L., Wang Y., Zhang P., Zhu M., Dong H. (2024). Recent Progress and Challenges of Photocatalytic CO_2_ Conversion into Value-Added Multi-Carbon Products. Coord. Chem. Rev..

[20] Kumagai H., Tamaki Y., Ishitani O. (2022). Photocatalytic Systems for CO_2_ Reduction: Metal-Complex Photocatalysts and Their Hybrids with Photofunctional Solid Materials. Acc. Chem. Res..

[21] Foorginezhad S., Ji X. (2025). Deep Eutectic Solvent-Based Slurry for CO_2_ Capture: Enhanced Efficiency and Kinetics. J. CO_2_ Util..

[22] Singh S., Verma R., Kaul N., Sa J., Punjal A., Prabhu S., Polshettiwar V. (2023). Surface Plasmon-Enhanced Photo-Driven CO_2_ Hydrogenation by Hydroxy-Terminated Nickel Nitride Nanosheets. Nat. Commun..

[23] Shen X., Wang Z., Guo H., Lei Z., Liu Z., Wang L. (2023). Solvent Engineering of Oxygen-Enriched Carbon Dots for Efficient Electrochemical Hydrogen Peroxide Production. Small.

[24] Jiang W., Loh H., Low B.Q.L., Zhu H., Low J., Heng J.Z.X., Tang K.Y., Li Z., Loh X.J., Ye E. (2023). Role of Oxygen Vacancy in Metal Oxides for Photocatalytic CO_2_ Reduction. Appl. Catal. B Environ..

[25] Shen Q., Lu Z., Bi F., Fang Y., Song L., Yang Y., Wu M., Zhang X. (2023). Effect of Actual Working Conditions on Catalyst Structure and Activity for Oxidation of Volatile Organic Compounds: A Review. Fuel.

[26] Li D., Kassymova M., Cai X., Zang S.-Q., Jiang H.-L. (2020). Photocatalytic CO_2_ Reduction over Metal-Organic Framework-Based Materials. Coord. Chem. Rev..

[27] Bi F., Ma S., Gao B., Liu B., Huang Y., Qiao R., Zhang X. (2024). Boosting Toluene Deep Oxidation by Tuning Metal-Support Interaction in Mof-Derived Pd@ ZrO_2_ Catalysts: The Role of Interfacial Interaction between Pd and ZrO_2_. Fuel.

[28] Ma X., Liu H., Yang W., Mao G., Zheng L., Jiang H.-L. (2021). Modulating Coordination Environment of Single-Atom Catalysts and Their Proximity to Photosensitive Units for Boosting Mof Photocatalysis. J. Am. Chem. Soc..

[29] Zhang C., Xie C., Gao Y., Tao X., Ding C., Fan F., Jiang H.L. (2022). Charge Separation by Creating Band Bending in Metal–Organic Frameworks for Improved Photocatalytic Hydrogen Evolution. Angew. Chem..

[30] Wang X., Zhu L., Lv Z., Qi Z., Xu Y., Miao T., Fu X., Li L. (2022). Coupled Visible-Light Driven Photocatalytic Reactions over Porphyrin-Based Mof Materials. Chem. Eng. J..

[31] Yue X., Cheng L., Li F., Fan J., Xiang Q. (2022). Highly Strained Bi-Mof on Bismuth Oxyhalide Support with Tailored Intermediate Adsorption/Desorption Capability for Robust CO_2_ Photoreduction. Angew. Chem. Int. Ed..

[32] Dong Y.-L., Liu H.-R., Wang S.-M., Guan G.-W., Yang Q.-Y. (2023). Immobilizing Isatin-Schiff Base Complexes in NH_2_-UiO-66 for Highly Photocatalytic CO_2_ Reduction. ACS Catal..

[33] Sun D., Kim D.-P. (2020). Hydrophobic Mofs@ Metal Nanoparticles@ Cofs for Interfacially Confined Photocatalysis with High Efficiency. ACS Appl. Mater. Interfaces.

[34] Mo Q., Zhang L., Li S., Song H., Fan Y., Su C.-Y. (2022). Engineering Single-Atom Sites into Pore-Confined Nanospaces of Porphyrinic Metal–Organic Frameworks for the Highly Efficient Photocatalytic Hydrogen Evolution Reaction. J. Am. Chem. Soc..

[35] Shang Y., Fan H., Yang X., Dong W., Wang W. (2023). Synergism between Chemisorption and Unique Electron Transfer Pathway in S-Scheme AgI/g-C_3_N_4_ Heterojunction for Improving the Photocatalytic H_2_ Evolution. J. Colloid Interface Sci..

[36] Anus A., Park S. (2024). The Synthesis and Key Features of 3d Carbon Nitrides (C_3_N_4_) Used for CO_2_ Photoreduction. Chem. Eng. J..

[37] Hu D.-D., Guo R.-T., Li C.-F., Yan J.-S., Pan W.-G. (2025). Construction of Indirect Dual S-Scheme Heterojunction CN/AP/AW Mediated by Ag Nanoparticles Enables Efficient CO_2_-to-CH_4_ Photoreduction. Sep. Purif. Technol..

[38] Li H., Gong H., Jin Z. (2022). Phosphorus Modified Ni-Mof–74/Bivo4 S-Scheme Heterojunction for Enhanced Photocatalytic Hydrogen Evolution. Appl. Catal. B Environ. Energy.

[39] Sayed M., Zhu B., Kuang P., Liu X., Cheng B., Ghamdi A.A.A., Wageh S., Zhang L., Yu J. (2022). Epr Investigation on Electron Transfer of 2d/3d g-C_3_N_4_/ZnO S-Scheme Heterojunction for Enhanced CO_2_ Photoreduction. Adv. Sustain. Syst..

[40] Javad Kalbasi R., Parishani P., Mazaheri O. (2018). Encapsulation of Nickel Nanoparticles and Homopoly (Vinylsulfonic Acid) in Mesoporous Carbon Cmk-3 as an Acid–Metal Bifunctional Catalyst for Tandem Reductive Amination. J. Clust. Sci..

[41] Maiti S., Pramanik A., Manju U., Mahanty S. (2015). Reversible Lithium Storage in Manganese 1, 3, 5-Benzenetricarboxylate Metal–Organic Framework with High Capacity and Rate Performance. ACS Appl. Mater. Interfaces.

[42] Sabir M., Sayed M., Zeng Z., Cheng B., Wang W., Wang C., Xu J., Cao S. (2025). Enhancing CO_2_ Photoreduction by Construction of g-C_3_N_4_/Co-Mofs S-Scheme Heterojunction. Appl. Surf. Sci..

[43] Yang J., Xiong P., Zheng C., Qiu H., Wei M. (2014). Metal–Organic Frameworks: A New Promising Class of Materials for a High Performance Supercapacitor Electrode. J. Mater. Chem. A.

[44] Maruthapandian V., Kumaraguru S., Mohan S., Saraswathy V., Muralidharan S. (2018). An Insight on the Electrocatalytic Mechanistic Study of Pristine Ni Mof (Btc) in Alkaline Medium for Enhanced Oer and Uor. ChemElectroChem.

[45] Yu Y., Huang H. (2023). Coupled Adsorption and Photocatalysis of g-C_3_N_4_ Based Composites: Material Synthesis, Mechanism, and Environmental Applications. Chem. Eng. J..

[46] Gallo E., Gorelov E., Guda A.A., Bugaev A.L., Bonino F., Borfecchia E., Ricchiardi G., Gianolio D., Chavan S., Lamberti C. (2017). Effect of Molecular Guest Binding on the D–D Transitions of Ni^2+^ of CPO-27-Ni: A Combined UV–Vis, Resonant-Valence-to-Core X-Ray Emission Spectroscopy, and Theoretical Study. Inorg. Chem..

[47] Zhao X., Xu M., Song X., Zhou W., Liu X., Huo P. (2022). 3d Fe-Mof Embedded into 2d Thin Layer Carbon Nitride to Construct 3d/2d S-Scheme Heterojunction for Enhanced Photoreduction of CO_2_. Chin. J. Catal..

[48] Li G., Sun Y., Zhang Q., Gao Z., Sun W., Zhou X. (2021). Ag Quantum Dots Modified Hierarchically Porous and Defective TiO_2_ Nanoparticles for Improved Photocatalytic CO_2_ Reduction. Chem. Eng. J..

[49] Kim D., Yong K. (2021). Boron Doping Induced Charge Transfer Switching of a C_3_N_4_/ZnO Photocatalyst from Z-Scheme to Type II to Enhance Photocatalytic Hydrogen Production. Appl. Catal. B Environ..

[50] Dong Y.L., Jiang Y., Ni S., Guan G.W., Zheng S.T., Guan Q., Pei L.M., Yang Q.Y. (2024). Ligand Defect-Induced Active Sites in Ni-Mof-74 for Efficient Photocatalytic CO_2_ Reduction to CO. Small.

[51] Xia P., Cao S., Zhu B., Liu M., Shi M., Yu J., Zhang Y. (2020). Designing a 0d/2d S-Scheme Heterojunction over Polymeric Carbon Nitride for Visible-Light Photocatalytic Inactivation of Bacteria. Angew. Chem. Int. Ed..

[52] Cheng C., Zhang J., Zhu B., Liang G., Zhang L., Yu J. (2023). Verifying the Charge-Transfer Mechanism in S-Scheme Heterojunctions Using Femtosecond Transient Absorption Spectroscopy. Angew. Chem. Int. Ed..

[53] Xia Y., Zhu B., Qin X., Ho W., Yu J. (2023). Zinc Porphyrin/g-C_3_N_4_ S-Scheme Photocatalyst for Efficient H_2_O_2_ Production. Chem. Eng. J..

[54] Pan J., Wang D., Zhang B., Zhao C., Liu D., Liu S., Zeng Z., Chen T., Liu G., Jiao S. (2024). Atomic-Level Charge Separation Boosting the Photocatalytic Hydrogen Evolution. Chem. Eng. J..

[55] Wang X., Liu B., Ma S., Zhang Y., Wang L., Zhu G., Huang W., Wang S. (2024). Induced Dipole Moments in Amorphous ZnCdS Catalysts Facilitate Photocatalytic H_2_ Evolution. Nat. Commun..

[56] Wang W., Liu Y., Chen S. (2024). Use of NiFe Layered Double Hydroxide as Electrocatalyst in Oxygen Evolution Reaction: Catalytic Mechanisms, Electrode Design, and Durability. Acta Phys. Chim. Sin..

[57] Kresse G., Hafner J. (1994). Ab initio molecular-dynamics simulation of the liquid-metal–amorphous-semiconductor transition in germanium. Phys. Rev. B.

[58] Kresse G., Furthmüller J. (1996). Efficient iterative schemes for ab initio total-energy calculations using a plane-wave basis set. Phys. Rev. B.

[59] Kresse G., Joubert D. (1999). From ultrasoft pseudopotentials to the projector augmented-wave method. Phys. Rev. B.

[60] Hammer B., Hansen L.B., Nørskov J.K. (1999). Improved adsorption energetics within density-functional theory using revised Perdew-Burke-Ernzerhof functionals. Phys. Rev. B.

[61] Monkhorst H.J., Pack J.D. (1976). Special points for Brillouin-zone integrations. Phys. Rev. B.

[62] Grimme S. (2006). Semiempirical GGA-type density functional constructed with a long-range dispersion correction. J. Comput. Chem..

[63] Dudarev S.L., Botton G.A., Savrasov S.Y., Humphreys C., Sutton A.P. (1998). Electron-energy-loss spectra and the structural stability of nickel oxide: An LSDA+ U study. Phys. Rev. B.

[64] Song K., Liang S., Zhong X., Wang M., Mo X., Lei X., Lin Z. (2022). Tailoring the crystal forms of the Ni-MOF catalysts for enhanced photocatalytic CO_2_-to-CO performance. Appl. Catal. B Environ..

[65] Han B., Ou X., Deng Z., Song Y., Tian C., Deng H., Xu Y.J., Lin Z. (2018). Nickel metal–organic framework monolayers for photoreduction of diluted CO_2_: Metal-node-dependent activity and selectivity. Angew. Chem. Int. Ed..

[66] Xu M., Sun C., Zhao X., Jiang H., Wang H., Huo P. (2022). Fabricated hierarchical CdS/Ni-MOF heterostructure for promoting photocatalytic reduction of CO_2_. Appl. Surf. Sci..

[67] Jiang J.-J., Li Y.-R., Zhang F.-J., Wang Y.-R. (2023). Novel honeycomb-like Ni-MOF enhanced hierarchical Bi2MoO6 microspheres for high efficient photocatalytic CO_2_ reduction. Inorg. Chem. Commun..

[68] Ali R.N., Qureshi W.A., Naz H., Jiang H., Yaseen M., Yu X., Liu Q. (2023). Synthesis of a highly active core–shell Ni-MOF@ CdS S-scheme heterojunction for enhanced photoreduction of CO_2_ to CO. New J. Chem..

[69] Wang Y., Luo Y., Yu S., Qin W., Xie Y. (2024). Organic-inorganic hybridization strategy for promoting BiOBr CO_2_ photoreduction via enhanced CO_2_ adsorption and photogenerated carrier migration. J. Catal..

[70] He B., Wang Y.-J., Bai X., Bian H., Xie Y., Li R., Li J.-R. (2024). Rational construction of MOF-on-MOF heterojunction with an array of flexible two-dimensional microsheets for efficient CO_2_ photoreduction. Chem. Eng. J..

[71] Chen Q., Li S., Xu H., Wang G., Qu Y., Zhu P., Wang D. (2020). Co-MOF as an electron donor for promoting visible-light photoactivities of g-C_3_N_4_ nanosheets for CO_2_ reduction. Chin. J. Catal..

