# Supplementary material for: Ni-MOF/g-C3N4 S-Scheme Heterojunction for Efficient Photocatalytic CO2 Reduction
Source: Materials (Basel). 2025 Jul 21;18(14):3419. doi: 10.3390/ma18143419 (PMC12300081; doi:10.3390/ma18143419)
Supplement: Supplementary file 1 [file materials-18-03419-s001.zip › materials-3736159-supplementary.pdf]

Supporting Information

Ni-MOF/g-C_3N_4 S-scheme Heterojunction for Efficient Photocatalytic CO_2 Reduction

Muhammad Sabir [1], Mahmoud Sayed [2,3], Iram Riaz [4], Guogen Qiu [1], Muhammad Tahir [5, *], Khuloud A. Alibrahim [6], Wang Wang [1,7, *]

1 State Key Laboratory of Advanced Technology for Materials Synthesis and Processing, Wuhan University of Technology, Wuhan 430070, China; m.sabir@whut.edu.cn; qgg@whut.edu.cn; doublewang@whut.edu.cn
2 Laboratory of Solar Fuel, Faculty of Materials Science and Chemistry, China University of Geosciences, Wuhan 430078, China
3 Chemistry Department, Faculty of Science, Fayoum University, Fayoum 63514, Egypt; msk07@fayoum.edu.eg
4 Department of coatings and polymeric materials, North Dakota State University, Fargo, ND 58102 US; iram.riaz@ndsu.edu
5 School of Mechatronical Engineering, Beijing Institute of Technology, Beijing 100081, P. R. China; tahir@bit.edu.cn
6 Department of Chemistry, College of Science, Princess Nourah bint Abdulrahman University, Riyadh 11671, Saudi Arabia; Kaalibrahim@pnu.edu.sa
7 Key Laboratory of Advanced Energy Materials Chemistry (Ministry of Education), Nankai University, Tianjin 300071, China
* Correspondence: doublewang@whut.edu.cn (Wang Wang)

Characterizations

X-ray diffraction (XRD) patterns were obtained using an X-ray diffractometer (D/Max-RB, Rigaku, Japan). Fourier transform infrared (FTIR) spectra were collected on a spectrometer (Nicolet iS50, Thermo Scientific, USA). Morphology and microstructure were observed using field-emission scanning electron microscopy (FESEM) (JSM-7500F, JEOL, Japan), and a transmission electron microscope (TEM) (Titan G2, FEI, USA). The thermogravimetric analysis was conducted using a DTG-60 instrument manufactured by SHIMADZU in Japan. The analysis was performed under ambient conditions, with a heating and cooling rate of 10 °C per minute, covering a temperature range from 30 to 600 ^{o}C. A UV-vis spectrophotometer (UV2600, Shimadzu, Japan) was used for obtaining the UV-vis diffuse reflectance spectra (UV-vis DRS). The photoluminescence (PL) spectroscopy was carried out using a fluorescence spectrophotometer (F-7000, Hitachi, Japan). Time-resolved photoluminescence (TRPL) spectra were measured using a fluorescence lifetime spectrophotometer (FLS 1000, Edinburgh, UK) with an excitation wavelength of 300 nm. Electron paramagnetic resonance (EPR) measurements were carried out using an ESR spectrometer (MEX-nano, Bruker) with a modulation frequency of 100 kHz and a microwave power of 15 mW. The electron spectrometer (AXIS Supra, Shimadzu, Japan) was used to acquire the in-situ X-ray photoelectron spectroscopy (XPS) spectra under light irradiation at λ=365 nm.

Photoelectrochemical measurements

The transient photocurrent response (i-t), electrochemical impedance spectra (EIS), and Mott-Schottky tests of g-C_3N_4 (CN) and Ni-MOF were investigated on electrochemical workstation (CCHI660C Instruments, China.). These experiments were conducted in a 0.5 M Na_2SO_4 aqueous solution, using a typical three-electrode setup. The reference, counter electrode, and working electrodes were, respectively, a Pt plate, a saturated Ag/AgCl, and fluorine-doped tin oxide (FTO) glass coated with the sample. First, 5 mg of the powder sample was completely disseminated in 20 μL of 5% Nafion solution (D-520, DuPont, USA) with 0.2 mL ethanol to prepare the working electrode. Subsequently, the blend was uniformly spun onto the conductive FTO glass surface (1.0 cm x 1.0 cm) and allowed to air dry for an entire night at ambient temperature. Additionally, during the i–t measurements, visible light irradiation was employed as the light source. In addition, for the i-t curves and EIS experiments, the applied potentials of 0.5 V and 1.5 V respectively. Mott-Schottky tests were conducted using potentials ranging from 1V to -1 V at 1000, and 2000 Hz frequencies.

Photocatalytic test

An experiment using photocatalysis was conducted in a 100-millilitre Pyrex flask. To be specific, 10 mg of the powders and 10 mg Ru as made were mixed with 50 mL of an aqueous solution containing 40 mL acetonitrile, 5 mL TEOA, and 5 mL H_2O. The photocatalytic reaction was conducted using an online analysis system (Lab Solar-6A, Beijing Perfect Light) connected to a gas chromatograph (GC2030, Shimadzu). After the suspension was degassed to eliminate any dissolved air, it was exposed to radiation using a 300 W Xe lamp equipped with AM1.5 G filter (100 mW/cm^2) (Beijing Perfect Light, Microsolar 300), and kept under 80 kPa CO_2 (99.999%). The light was placed at a distance of 10 cm from the flask. The injection rate is 1.5 kPa/s. To maintain the reaction mixture at room temperature, water was pumped through a glass jacket to cool the reactor. Using helium carrier gas (99.999%), an online gas chromatograph equipped with a barrier discharge ionization detector (BID) was able to determine the quantity of gas product.

DFT calculations

The Vienna Ab-initio Simulation Package was used to perform DFT calculations (VASP) and using the projector augment wave (PAW) [1-3]. Specifically, C $2s^2 2p^2$, N $2s^2 2p^3$, H $1s^1$, and Co $3d^8 4s^1$ states were used as the valence configurations. The exchange and correlation potential was described using the generalized gradient approximation (GGA) using Perdew-Burke-Ernzerh of (PBE) [4]. For the plane wave basis set, 450 eV was chosen as the cutoff energy [5]. A Brillouin zone sample was employed. Above the Ni-MOF/CN heterostructure, a 12-Å vacuum area was created to reduce the interactions between nearby systems. Geometry optimizations were carried out until the force on each ion was less than 0.05 eV/Å, and the total system energy was minimized to below 1×10^{-5} eV. The van der Waals interactions were accounted for using the DFT-D3(BJ) method proposed by Grimme [6]. The DFT+U method was adopted for geometry optimization, and the effective on-site Coulomb interaction parameters of 5 eV was applied to the Ni-3*d* electrons [7]. Spin polarized calculation was used throughout all calculations.

Calculation of lifetime of fluorescence

The decay curves obtained from TRPL can be well fitted by the following three-exponential equation:

$$I_{(t)} = I_{(0)} + A_1 \exp(-t/\tau_1) + A_2 \exp(-t/\tau_2) + A_3 \exp(-t/\tau_3) \qquad (1)$$

Where the I_0 represents the baseline correction value, A1, A2, and A3 are the pre-exponential factors, and τ_1, τ_2 represent the lifetime of the radiant energy transfer process (ns), while τ_3 represents the lifetime of the non-radiative energy transfer process (ns). The average lifetime (τ_{ave}) can be calculated according to the following equation:

$$\tau_{ave} = (A_1\tau_1^2 + A_2\tau_2^2 + A_3\tau_3^2) / (A_1\tau_1 + A_2\tau_2 + A_3\tau_3) \qquad (2)$$

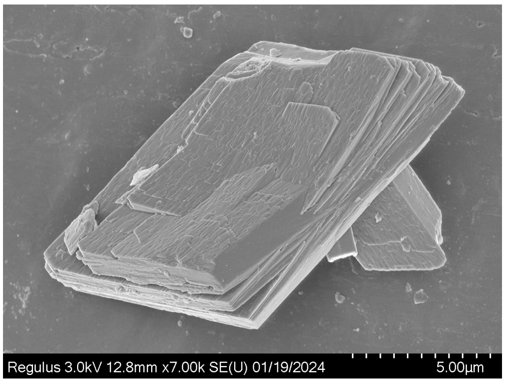

Figure S1. FESEM of Ni-MOF.

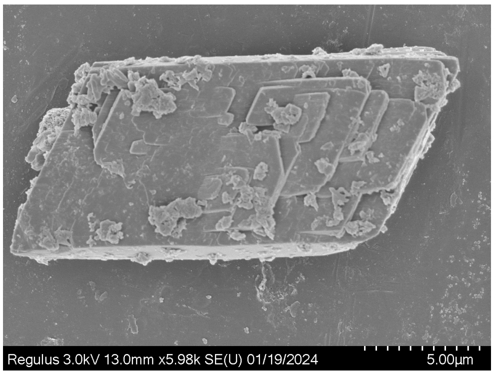

Figure S2. FESEM of CN/NMF.

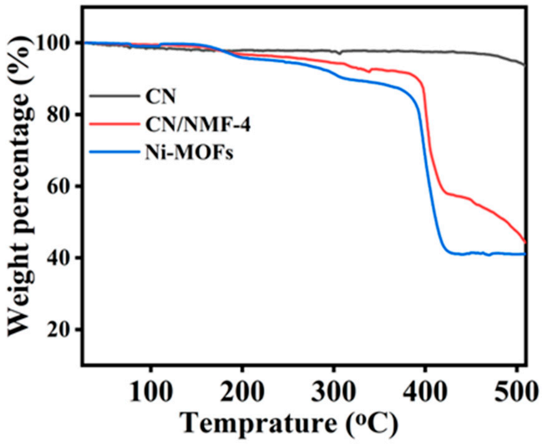

Figure S3. TGA analysis of Ni-MOF, CN and CN/NMF-4.

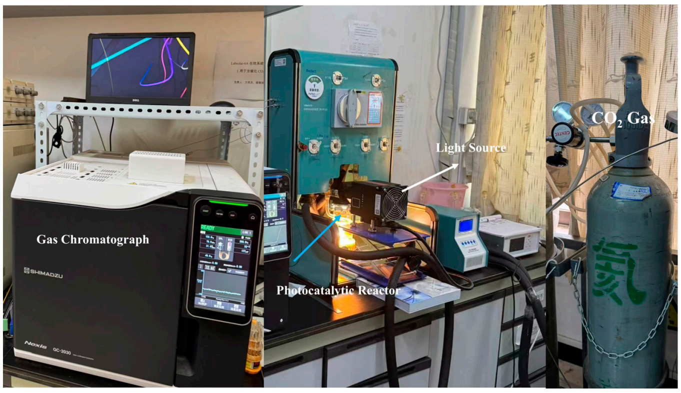

Figure S4. Image of the on-line photocatalytic CO_2 reduction system.

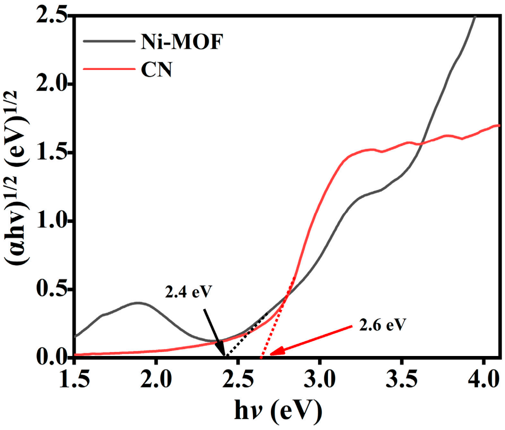

Figure S5. Tauc plot of Ni-MOF, and CN.

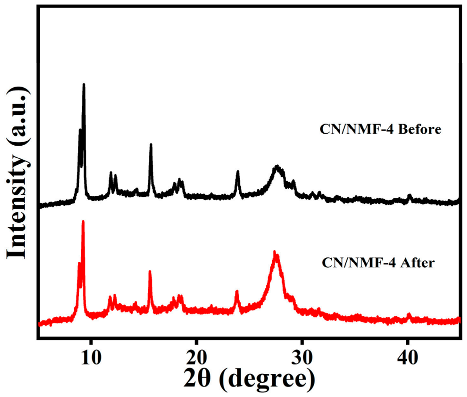

Figure S6. XRD of CN/NMF-4 before and after three cycles

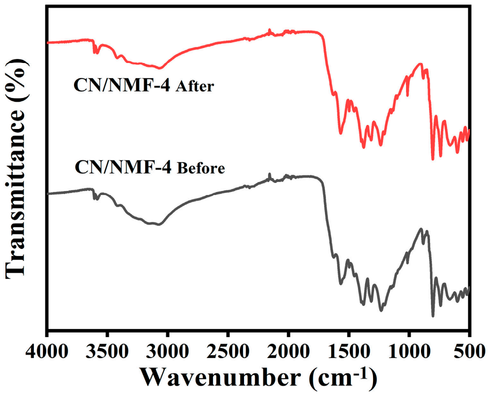

Figure S7. FTIR of CN/NMF-4 before and after three cycles

Table. S1. Ni-MOFs and their composites based photocatalysts for CO_2 reduction.

Catalysts	Condition	Light	Product	Yield μmol h^{-1} g^{-1}	Ref..
**Ni-MOF**	7.5 mg Ru, 1.0 mg catalyst, 3 mL acetonitrile,2 mL H_2O, and 1 mL (TEOA)	$\lambda \geq 400$	CO	34	[8]
**Ni-MOF-74**	60 mg Ru, 4 mL TEOA,60 mL acetonitrile, 4 mL H_2O,3mg of catalyst, 3 mL	$\lambda \geq 400$	CO	1380	[9]
**Ni-MOF**	1 mg catalyst, Ru, 7.5 mg, 2 mL H_2O, 3 mL acetonitrile, and 1 mL TEOA	400 nm $\leq \lambda \leq$ 800	CO	12.5	[10]
**Ni-MOF/CdS**	50 mg catalyst, 40 mL acetonitrile ,5 mL water	300 W Xe lamp	CO	7.47	[11]
**Ni-MOF/Bi_2MoO_6**	10 mg catalyst,5 mL of water	300 W Xe lamp	CO	66.34	[12]
**Ni-MOF/CdS**	20 mg, catalyst, 50 mL of H_2O	300 W Xe lamp	CO	24.1	[13]
**BiOBr/Ni-MOF**	5 mg catalyst, 5 mg Ru, 6 ml acetonitrile,6 ml TEOA,6 ml Water	300 W Xe lamp	CO	121.8	[14]
**NH_2-MIL-125@Ni-BDC**	1 mg catalyst,100 μL TEA, 100 μL H_2O	300 W Xe lamp	CO	41.38	[15]
**Fe-MOF/g-CN**	100 mL H_2O	300 W Xe lamp	CO	**6.39**	[16]
**Co-MOFs/g-CN**	10 mg catalyst, 40 mL Acetonitrile, 5 mL H_2O 5mL TEOA	300 W Xe lamp	CO	16.1	[17]
**Co-MOFs/g-CN**	20 mg catalyst,4 mL water	300 W Xe lamp	CO	6.05	[18]
**Ni-MOF/CN**	**10 mg, Ru,10 mg cat, 40 mL Acetonitrile, 5 mL H_2O 5mL TEOA**	300 W Xe lamp	**CO**	**1014.6**	**This work**

Table. S2. Fitting parameters of time-resolved photoluminescence spectra of CN, Ni-MOF and CN/NMF-4 samples.

Sample	τ_1(ns)	Rel.%	τ_2(ns)	Rel.%	τ_3(ns)	Rel.%	τ_{ave} (ns)
CN	1.2	21.25	4.14	48.59	20.18	30.16	8.36
Ni-MOF	0.39	47.74	3.18	38.89	16.70	13.37	3.65
CN/NMF-4	1.07	22.61	3.89	57.23	17.63	20.15	6.01

Table. S3. Thermal gravimetric analysis (TGA) data. CN, Ni-MOF and CN/NMF-4 samples.

Sample	Weight loss region I Temp (°C)	Weight loss region I wt%	Weight loss region II Temp (°C)	Weight loss region II wt%	Ratio of wt% I/II
CN	~100–500 (very slight)	~2%	–	–	–
CN/NMF-4	~50–330	~10%	~330–460	~45%	~0.22
Ni-MOF	~50–300	~15%	~300–450	~45%	~0.33

References

1. Kresse, Georg, and Jürgen Hafner. Ab Initio Molecular-Dynamics Simulation of the Liquid-Metal–Amorphous-Semiconductor Transition in Germanium. *Phys. Rev. B* 49, no. 20 (1994): 14251.
2. Kresse, Georg, and Jürgen Furthmüller. Efficient Iterative Schemes for Ab Initio Total-Energy Calculations Using a Plane-Wave Basis Set. *Phys. Rev. B* 54, no. 16 (1996): 11169.
3. Kresse, Georg, and Daniel Joubert. "From Ultrasoft Pseudopotentials to the Projector Augmented-Wave Method. *Phys. Rev. B* 59, no. 3 (1999): 1758.
4. Hammer, BHLB, Lars Bruno Hansen, and Jens Kehlet Nørskov. Improved Adsorption Energetics within Density-Functional Theory Using Revised Perdew-Burke-Ernzerhof Functionals. *Phys. Rev. B* 59, no. 11 (1999): 7413.
5. Monkhorst, Hendrik J, and James D Pack. Special Points for Brillouin-Zone Integrations. *Phys. Rev. B* 13, no. 12 (1976): 5188.
6. Grimme, Stefan. Semiempirical Gga-Type Density Functional Constructed with a Long-Range Dispersion Correction. *J. Computat. Chem.* 27, no. 15 (2006): 1787-99.
7. Dudarev, Sergei L, Gianluigi A Botton, Sergey Y Savrasov, CJ Humphreys, and Adrian P Sutton.

Electron-Energy-Loss Spectra and the Structural Stability of Nickel Oxide: An Lsda+ U Study. *Phys. Rev. B* 57, no. 3 (1998): 1505.
8. Song, Kainan, Shujie Liang, Xiaohui Zhong, Mengye Wang, Xiaofeng Mo, Xueqian Lei, and Zhang Lin. Tailoring the Crystal Forms of the Ni-Mof Catalysts for Enhanced Photocatalytic CO_2-to-CO Performance. *Appl. Catal. B: Environ.* 309 (2022): 121232.
9. Dong, Yong-Li, Yu Jiang, Shuang Ni, Guo-Wei Guan, Su-Tao Zheng, Qingqing Guan, Ling-Min Pei, and Qing-Yuan Yang. Ligand Defect-Induced Active Sites in Ni-Mof-74 for Efficient Photocatalytic CO_2 Reduction to Co. *Small* 20, no. 23 (2024): 2308005.
10. Han, Bin, Xinwen Ou, Ziqi Deng, Yao Song, Chen Tian, Hong Deng, Yi-Jun Xu, and Zhang Lin. "Nickel Metal–Organic Framework Monolayers for Photoreduction of Diluted CO_2: Metal-Node-Dependent Activity and Selectivity. *Angew Chemie Int. Ed.* 57, no. 51 (2018): 16811-15.
11. Xu, Mengyang, Chao Sun, Xiaoxue Zhao, Haopeng Jiang, Huiqin Wang, and Pengwei Huo. Fabricated Hierarchical Cds/Ni-Mof Heterostructure for Promoting Photocatalytic Reduction of CO_2. *Appl. Sur. Sci.* 576 (2022): 151792.
12. Jiang, Jing-Jing, Ya-Ru Li, Feng-Jun Zhang, and Ying-Rui Wang. Novel Honeycomb-Like Ni-Mof Enhanced Hierarchical Bi_2MOO_6 Microspheres for High Efficient Photocatalytic CO_2 Reduction. *Inorg. Chem. Commun.* 156 (2023): 111271.
13. Ali, Rai Nauman, Waqar Ahmad Qureshi, Hina Naz, Haopeng Jiang, Maria Yaseen, Xiaohui Yu, and Qinqin Liu. Synthesis of a Highly Active Core–Shell Ni-Mof@ Cds S-Scheme Heterojunction for Enhanced Photoreduction of CO_2 to Co. *New J. Chem.* 47, no. 33 (2023): 15534-42.
14. Wang, Yiqiao, Yongping Luo, Shuohan Yu, Wenzhen Qin, and Yu Xie. "Organic-Inorganic Hybridization Strategy for Promoting Biobr CO_2 Photoreduction Via Enhanced CO_2 Adsorption and Photogenerated Carrier Migration. *Journal of Catalysis* 429 (2024): 115295.
15. He, Bing, Ying-Jie Wang, Xuefeng Bai, He Bian, Yabo Xie, Rui Li, and Jian-Rong Li. Rational Construction of Mof-on-Mof Heterojunction with an Array of Flexible Two-Dimensional Microsheets for Efficient CO_2 Photoreduction. *Chem. Eng. J.* 482 (2024): 149000.
16. Zhao, Xiaoxue, Mengyang Xu, Xianghai Song, Weiqiang Zhou, Xin Liu, and Pengwei Huo. 3d Fe-Mof Embedded into 2d Thin Layer Carbon Nitride to Construct 3d/2d S-Scheme Heterojunction for Enhanced Photoreduction of CO_2.*Chin. J. Catal.* 43, no. 10 (2022): 2625-36.
17. Sabir, Muhammad, Mahmoud Sayed, Zhuofan Zeng, Bei Cheng, Wang Wang, Chuanbin Wang, Jingsan Xu, and Shaowen Cao. Enhancing CO_2 Photoreduction by Construction of g-C_3N_4/Co-Mofs S-Scheme Heterojunction. *Appl. Sur. Sci.*693 (2025): 162752.
18. Chen, Qiuyu, Sijia Li, Hongyi Xu, Guofeng Wang, Yang Qu, Peifen Zhu, and Dingsheng Wang. Co-Mof as an Electron Donor for Promoting Visible-Light Photoactivities of g-C_3N_4 Nanosheets for Co2 Reduction. *Chin. J. Catal.* 41, no. 3 (2020): 514-23.
